# Protective effects of curcumin against chemical-induced toxicity in the male reproductive system: a systematic review

**DOI:** 10.3389/fphar.2026.1749370

**Published:** 2026-01-29

**Authors:** Mitra Tarlan, Nillofar Heidrizadeh, Sara Gooharpoor, Omid Tavallaei, Saeed Khazayel, Mohamad Hosein Farzaei, Javier Echeverría

**Affiliations:** 1 Pharmaceutical Sciences Research Center, Health Institute, Kermanshah University of Medical Sciences, Kermanshah, Iran; 2 Departamento de Ciencias del Ambiente, Facultad de Química y Biología, Universidad de Santiago de Chile, Santiago, Chile

**Keywords:** antioxidant, chemical toxicity, curcumin, inflammation, systematic review

## Abstract

**Background:**

Curcumin is a biologically active substance present in turmeric. It has recently been suggested for its protective potential against a wide variety of chemical-induced toxicities.

**Purpose:**

This systematic review aims to summarize current evidence on the protective effects of curcumin against chemical-induced toxicity, with particular emphasis on its impact on the male reproductive system.

**Methods:**

A literature search was conducted using the major databases PubMed®, Scopus®, Web of Science®, and ScienceDirect®, up to December 2024. This review encompassed studies assessing curcumin’s protective role against chemical toxicity, both *in vitro* and *in vivo*. Extracted data included the type of chemical agent, dosage, curcumin formulation, and reported toxicity outcomes.

**Results and discussion:**

A total of 31 studies were included in the present review based on the established inclusion criteria. The toxicants studied contained heavy metals (lead and cadmium), pesticides (e.g., Malathion), and industrial solvents (notably titanium dioxide nanoparticles). Curcumin has demonstrated significant protective effects through multiple mechanisms, including antioxidant activity, anti-inflammatory effects, and modulation of detoxification enzymes. Interestingly, curcumin supplementation was associated with reduced oxidative stress markers and improved histopathological findings across various animal models. The effective dose varied widely across studies, with most showing positive effects at doses between 50 mg/kg and 200 mg/kg.

**Conclusion:**

The results of this systematic review suggest that curcumin holds promise for preventing various chemical-induced toxicities. Its diversified mechanisms of action show promise as a therapeutic agent for the relief of chemical toxicity. Nonetheless, additional studies are required to determine the most effective dosing strategies, examine bioavailability, and assess the safety of long-term use.

## 1 Introduction

### 1.1 Background

The male reproductive system is highly vulnerable to environmental and chemical agents, which can result in substantial health issues, including infertility. Chemical-induced toxicity has been recognized as an alarming challenge, and in particular, the exposure of men to industrial chemicals, drugs, and environmental pollutants. These toxic agents can disrupt normal reproductive physiology by altering hormonal balance, spermatogenesis, sperm motility, and morphology. Toxicity against the male reproductive system is driving a need for understanding the mechanistic basis for possible protective strategies. Recent research has highlighted the role of antioxidants in mitigating these toxic effects and has suggested natural agents, such as curcumin, as potential treatments for reproductive health (Riahi et al., 2021).

Oxidative stress plays a pivotal role in both maintaining health and contributing to the development of various pathological conditions, particularly those associated with chemical-induced toxicity (Bustos et al., 2025). It arises from an imbalance between excessive reactive oxygen species (ROS) generation and the capacity of endogenous antioxidant defense systems, leading to lipid peroxidation, protein oxidation, DNA damage, and cellular dysfunction. Accumulating evidence indicates that oxidative stress is a primary underlying mechanism in male reproductive disorders, including testicular damage, impaired spermatogenesis, hormonal dysregulation, and reduced fertility. Consequently, antioxidants have garnered considerable attention as protective agents that can restore redox homeostasis and mitigate toxicant-induced reproductive damage.

### 1.2 Chemical-induced toxicity on the male reproductive system

Chemical exposure induces a cascade of adverse effects on the male reproductive system (e.g., changes in hormone profiles, sperm production, and blood and semen quality), collectively defining male reproductive health. Contaminants, including heavy metals, pesticides, and some pharmaceuticals, have been shown to affect spermatogenesis and induce oxidative stress, with a crucial role in male subfertility. For instance, exposure to lead and cadmium has been linked with reduced sperm count and higher sperm abnormalities. Further, it has been demonstrated that chemicals can interfere with the endocrine function and lead to endocrine imbalance with an adverse impact on fertility. However, it is of great importance to understand the exact mechanisms through which these chemicals contribute to their toxicity, so that suitable protective measures can be implemented (Momeni and Eskandari, 2016).

### 1.3 Mechanisms of toxicity

The mechanisms by which chemicals are toxic to the male reproductive system involve multiple signaling pathways and are often accompanied by oxidative and inflammatory stress responses. ROS generated by chemical exposure within the cellular environment can inactivate cellular components (lipids, proteins, and DNA), thereby impacting spermatogenesis and sperm quality. In addition, numerous toxins disrupt the reproductive system’s antioxidant balance, thereby increasing oxidative stress. Inflammation arising from chemical exposure adds insult to injury by inducing apoptosis in germ cells and distorting hormonal signaling, which is crucial for normal reproductive performance. These findings reveal bidirectional crosstalk between oxidative stress and inflammation, underscoring the need for antioxidants to counteract these adverse effects (Hussain et al., 2024; Panga and Zhao, 2024).

### 1.4 Effects on reproductive indices

Chemical-induced toxicity can disturb many reproductive parameters, such as the number of spermatozoa, sperm motility, sperm morphology, and, in general, reproductive fertility rates. Studies show that exposure to certain toxins may reduce sperm count and viability while increasing the incidence of abnormal sperm shapes. For example, exposure to sodium arsenite has been demonstrated to decrease epididymal sperm count and motility in animal models. Additionally, hormonal profile changes caused by chemical exposure may also exacerbate reproductive issues through partial or complete impairment of testicular function and accessory gland activity. These adverse effects underline the urgent need for effective strategies to safeguard male reproductive health from chemical attacks (Sadoughi et al., 2019; Tsao et al., 2022).

### 1.5 Medicinal plants, oxidative stress, and natural therapeutic agents

Medicinal plants have long been recognized as rich sources of bioactive compounds with potent antioxidant and anti-inflammatory properties (Yeung et al., 2019). Phytochemicals derived from natural sources can modulate oxidative stress by scavenging free radicals, enhancing endogenous antioxidant enzyme activity, and regulating redox-sensitive signaling pathways (Wan et al., 2023). Among these compounds, curcumin and related curcuminoids have garnered considerable scientific interest due to their broad spectrum of biological activities (Aitken and Baker, 2006; Agarwal et al., 2014; Forero-Doria et al., 2022).

Curcumin has been shown to attenuate oxidative damage, suppress inflammatory mediators, and regulate apoptosis-related pathways in various experimental models. Similarly, curcumol, another bioactive compound isolated from plant roots, exhibits antioxidant and cytoprotective effects and has been reported to interfere with the progression of oxidative stress–related diseases (Wei et al., 2019). Furthermore, accumulating evidence suggests that curcumin can modulate oxidative stress, inflammation, nervous system function, and lipid metabolism, underscoring its therapeutic potential in preventing and managing oxidative stress–related disorders (Sureda et al., 2023).

These findings support the increasing interest in natural agents and medicinal plants as promising alternatives or complementary strategies for mitigating chemically induced toxicity and preserving reproductive health.

### 1.6 Role of curcumin

Curcumin, the major phytochemical of the rhizome of *Curcuma longa* L. [Zingiberaceae], is increasingly being studied for its chemopreventive properties in protecting against male reproductive system toxicity from chemical exposure (Tarlan et al., 2025). Its antioxidant activity helps fight free radicals, reduce oxidative stress, and prevent damage to germ cells caused by environmental toxins. Furthermore, curcumin exhibits anti-inflammatory properties that may blunt inflammatory responses induced by chemotrauma. Recent research has shown that curcumin improves sperm motility and viability and restores hormonal dysbalance caused by toxins in testicular function. This suggests that curcumin may be a promising therapeutic approach for male fertility preservation (Tsao et al., 2022).

### 1.7 Evidence from animal studies

Evidence from animal experiments indicates that curcumin helps guard the male reproductive organs against drug-related toxic effects. For example, curcumin has been shown to effectively attenuate the harmful effects of multiple toxicants on sperm parameters in rodents. In a study of sodium arsenite exposure, curcumin treatment increased sperm count and motility compared with controls. Moreover, curcumin has been found to upregulate the testicular antioxidant pretension and reduce testicular oxidative stress and inflammatory markers. These findings suggest the potential of curcumin as a protective agent against reproductive toxicity caused by environmental toxicants (Tsao et al., 2022).

### 1.8 Clinical implications

The implications of research on curcumin’s protective effects extend beyond animal evidence to include potential clinical applications for human health. Considering its known safety and bioactivity, curcumin supplementation may be worth investigating as an adjunct therapy in men chronically exposed to environmental contaminants or with impaired fertility associated with oxidative stress. Research in the form of current clinical trials is aimed at determining the potential of curcumin to enhance sperm parameters in males with oxidative damage–related infertility. If curcumin is found efficacious in larger human trials, it may be a beneficial adjunctive therapy to improve male reproductive health amid the backdrop of chemical exposures in today’s society (Riahi et al., 2021).

The necessity of this study has developed because of the growing incidence of male reproductive disorders resulting from chemical-induced toxicity, which represents a serious threat to male fertility worldwide. Given the numerous environmental and pharmaceutical agents known to affect reproductive health through oxidative stress and hormonal imbalance, the development of effective protective strategies is of great importance. The objectives of this systematic review are to critically assess the protective potential of curcumin and its nanoscale rendition, nanocurcumin, against drug-induced toxicity in the male reproductive system. To clarify the mechanisms by which curcumin may act as an antioxidant and anti-inflammatory agent to help offset the adverse effects of toxic exposures on reproductive indices, this review examines studies from animal and clinical trials. Overall, the current study aims to provide a framework for future investigations and clinical applications that may improve male reproductive health and fertility through natural therapeutic approaches.

## 2 Methods

### 2.1 Search strategy

**FIGURE 1:**
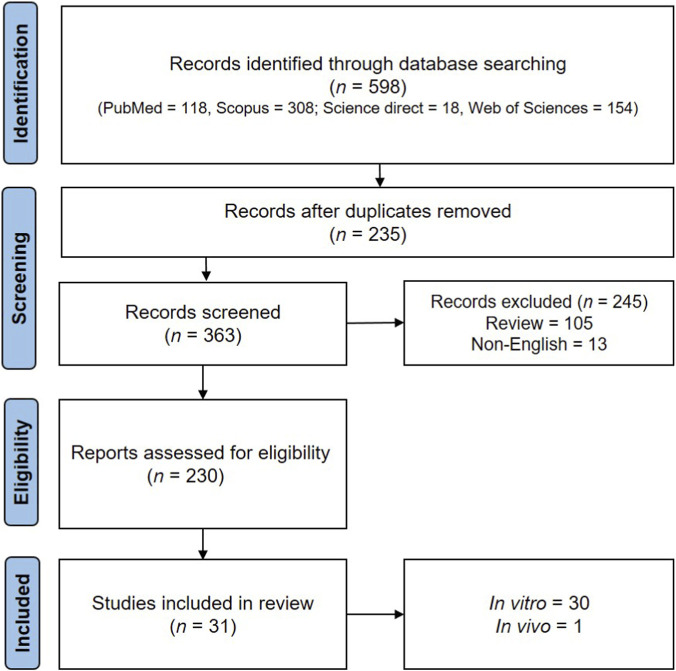
The PRISMA flowchart of the selection process of the included studies.

This systematic review was conducted in accordance with the Preferred Reporting Items for Systematic Reviews and Meta-Analyses (PRISMA) checklist (Moher, 2009). The following electronic databases were utilized: ScienceDirect®, PubMed®, Web of Science®, and Scopus®. The search was done with the following terms: ((male infertility OR male subfertility OR male sterility OR aspermia OR azoospermia OR sperm OR testicular tissue OR sex hormones OR semen OR oligozoospermia) AND (curcumin OR nano curcumin)).in the full text. Further details on the articles selected for the study are provided in the PRISMA diagram shown in Figure 1.

### 2.2 Inclusion criteria

Published articles in English reporting *in vivo* and *in vitro* experimental research on the effects of curcumin on chemical-induced toxicity in the male reproductive system were also considered.

### 2.3 Exclusion criteria

The exclusion criteria applied in this review were as follows: i) conference abstracts, books, book chapters, and unpublished studies; ii) articles written in languages other than English; iii) review articles, systematic reviews, meta-analyses, and letters; and iv) original research that did not use tumor cell lines or animal models.

### 2.4 Data extraction

The last 31 eligible studies were examined for phytochemical name, study type, model, intervention, and outcome. The article-level separation information is also shown in the PRISMA diagram below (Figure 1).

## 3 Results and discussion

### 3.1 The role of curcumin in mitigating chemical-induced reproductive toxicity in males

**FIGURE 2:**
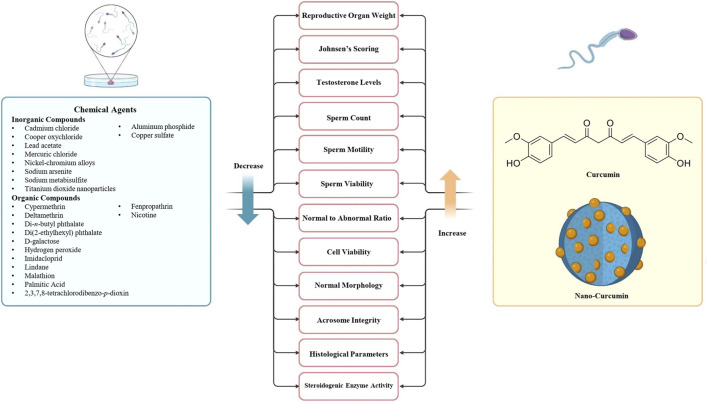
Investigating the adverse effects of chemical exposure on male reproductive health and the potential protective role of curcumin and nanocurcumin in mitigating toxicity.

This section highlights various studies suggesting that specific chemical agents may have adverse effects, including deterioration of spermatogenesis or hormonal imbalance. These adverse side effects can be mitigated by curcumin’s antioxidant and anti-inflammatory actions, which reduce oxidative stress and enhance cellular health. This section introduces the inorganic (Table 1) and organic (Table 2) chemical agent that affects the male reproductive system and analyzes the mechanisms by which curcumin exerts its protective effects, outlining its relevance as an adjuvant therapeutic approach. It highlights the need to exploit natural compounds, such as curcumin and its nanoformulations (Table 3), to safeguard male reproductive health during pharmacological treatment (Figure 2).

**TABLE 1:** The function of curcumin in reducing reproductive toxicity in males caused by inorganic chemicals.

Compound/preparation	Drug and doses	Model	Duration	Outcome	Reference
Curcumin (10 mg/animal/day)	Cadmium chloride, CdCl_2_ (50 mg/kg/animal/day)	Mice	15 days	↑ Cellular contact and associations in seminiferous tubules. • Slight histopathological damage. • Seminiferous tubules retained typical structures	Singh et al. (2012)
Curcumin (10 mg/animal/day)	Cadmium chloride, CdCl_2_ (50 mg/kg)	Mice	15 days	↓ Histopathological damage	Yang et al. (2019)
Curcumin (100 mg/kg)	Cadmium chloride, CdCl_2_ (1 mg/kg)	Rats	3 days	Mitigated oxidative stress by reducing TBARS levels ↑ SOD ↑ CAT ↑ GSHPX ↑ GSH ↑ Sperm motility ↑ Sperm concentration	Oguzturk et al. (2012)
Curcumin (100 mg/kg)	Cadmium chloride, CdCl_2_ (1 mg/kg)	Rats	4 weeks	Restoring the mean seminiferous tubule diameter and mean testicular biopsy score values ↑ Testosterone levels. ↓ Activity of *in situ* identification of apoptosis using terminal dUTP nick end-labeling	Aktas et al. (2012)
Curcumin (100 mg kg^−1^)	Cadmium chloride, CdCl_2_ (5 mg/kg)	Mice	34 h	↑ Total glutathione, ↑ Total thiol ↑ Diameter of seminiferous tubules. ↓ MDA ↓ H_2_O_2_	Momeni and Eskandari (2020)
Curcumin (80 mg/kg b.wt./day)	Copper oxychloride, COC (50,100, and 200 mg/kg)	Rats	90 days	↑ SOD ↑ CAT ↓ LPO Preserved the structure of the seminiferous tubules. ↓ Necrosis. Maintained germ cell integrity restored serum testosterone levels ↑ Sperm count. ↑ Sperm motility	Gad El-Hak and Mobarak (2020)
Curcumin (100, 200, and 400 mg/kg)	Lead acetate, Pb(OAc)_2_ (50 mg/kg)	Rats	40 days	Improved testicular damage, necrosis of seminiferous tubules, and loss of spermatid. ↑ Sperm count. ↑ Sperm motility. ↑ Sperm viability. ↑ SOD ↑ GSHPx. ↓ MDA (400 mg/kg)	Sudjarwo et al. (2017)
Curcumin (100 mg/kg body wt)	Pb (0.188 ppm), As (0.038 ppb), Cd (0.016 ppb), Hg (0.011 ppb), Fe (1.792 ppm), and Cu (1.67 ppm) metal mixture: 10×–100× environmental dose	Rats	28 days	↑ 17β-HSD ↑ ACP ↑ GGT ↑ LDH ↑ SDH ↓ MDA ↑ CAT ↑ SOD ↑ GSH ↓ Degeneration and histoarchitecture of testicular tissues. ↑ Cellular integrity within seminiferous tubules	Zoheb et al. (2014)
Curcumin (100 mg/kg)	Nickel (0.2 ppm) and chromium (0.5 ppm)	Rats	75 days	↑ GSHPx. ↑ GR ↑ CAT ↑ SOD	El-Amawy et al. (2022)
Curcumin (150 and 300 ppm)	Mercuric chloride, HgCl_2_ (10 ppm)	Mice	15 days	↑ FSH ↑ Testosterone ↑ Sperm count. ↑ Sperm motility. ↑ Viable sperm. Effect on sexual behavior	Mohammad Abu-Taweel (2020)
Curcumin (100 mg/kg)	Sodium arsenite, NaAsO_2_ (0.5 mg/kg)	Mice	5 week	↑ Sperm count. ↑ Sperm motility. ↑ Sperm viability. ↑ Normal morphology. ↑ Acrosome integrity	Momeni and Eskandari (2016)
Curcumin (100 mg/kg/day)	Sodium metabisulfite Na_2_S_2_O_5_ (7.70 mg/kg)	Rats	7 weeks	↑ Volume of seminiferous tubules. ↑ Tubular epithelium. ↑ Tubule length. ↑ Connective tissue volume	Mahmoudi et al. (2017)
Curcumin (200 mg/kg)	Titanium dioxide nanoparticle, nTiO_2_ (50 mg/kg)	Mice	7 days	↑ Testicular weight ↑ Johnsen’s scoring. ↑ Testosterone levels. ↑ Sperm count. ↑ Sperm motility. ↑ Percentage of abnormality. ↑ Histological criteria such as vacuolization, detachment, and sloughing of germ cells into the seminiferous tubules. ameliorated morphometric parameters	Karimi et al. (2019)
Curcumin (1, 10, and 100 mg/kg bw/1 mL)	Sodium fluoride (10 mg/kg) and fluoride-contaminated groundwater (5 mg F/L)	Rats	52 days	Prevent the decrease in body weight and relative weight. Attenuated the reduction in total sperm count, sperm motility, and abnormal sperm counts caused by NaF and fluoride exposure. Mitigated the decrease in the activity of testicular 3β-HSDH induced by NaF and fluoride exposure. Prevented the decline in serum testosterone levels. ↑ SOD ↑ CAT ↑ MDA Maintain the counts of different stages of spermatogonial cells	Chaithra and Sarjan (2020)

**TABLE 2:** The function of curcumin in reducing reproductive toxicity in males caused by organic chemicals.

Compound/ Preparation	Drug and doses	Model	Duration	Outcome	Reference
Curcumin (100 mg/kg)	Cypermethrin and Deltamethrin (2 mg/kg)	Rats	45 days	↑ Increasing reproductive organ weight. ↑ Sperm count. ↑ Sperm motility. Restoring testosterone, FSH, and LH levels. ↑ Upregulating steroidogenic enzyme activity	Sharma et al. (2018)
Curcumin (200 mg/kg/day)	di-n-butyl phthalate (DBP) (2 g/kg)	Rats	16 days	↑ GSH ↑ CAT ↑ G6PD ↑ SOD ↑ Spermatozoa motility ↓ Percentage of abnormal spermatozoa Recovery of the seminiferous tubules. Prevention of necrosis and defoliation of spermatocytes	Farombi et al. (2007)
Curcumin (300 mg/kg/day)	di-n-butyl phthalate (DBP) (500 mg/kg/day)	Rats	3 weeks	↓ Testicular ferroptosis. ↑ PRDX6	Bu et al. (2023)
Curcumin (1–100 μM)	di(2-ethylhexyl)phthalate (DEHP) (1,000 mg/kg/day)	Mice	30 days	Low concentrations of curcumin (1–50 μM): ↓ Sperm motility and ↓ Testicular damage. High concentrations (100 μM) of curcumin: ↓ Sperm motility	Głombik et al. (2014)
Curcumin (200 mg/kg)	H_2_O_2_ (1 mg/kg)	Rooster	Thirty-two 20-week roosters	↓ Abnormal sperm rates in the semen. ↑ Seminiferous tubules. ↑ Testis scores. ↑ Serum testosterone levels. ↑ Capacities of antioxidant enzymes (CAT, GSH-Px, SOD, and T-AOC) ↓ MDA levels. ↑ Spermatogenesis-related genes (STAR, HSD3-β1, SYCP3, AKT1) ↑ Antioxidant genes (HMOX-1, NQO-1)	Wu et al. (2023)
Curcumin (100 mg/kg, body weight)	Imidacloprid (45 and 90 mg/kg)	Rats	28 days	↑ Total epididymal sperm count. ↑ Sperm motility. ↑ Live sperm count. ↓ Sperm abnormalities. ↑ 3β-HSD ↑ 17β-HSD ↓ LPO ↑ GSH ↑ SOD ↑ GSHPx ↑ GST	Lonare et al. (2016)
Curcumin (100 mg/kgbw)	Lindane (30 mg/kg)	Rats	14 and 28 days	↓ The decrease in testes and cauda epididymis weight ↑ Sperm headcounts. ↓ Abnormal sperm morphology. ↑ SOD ↑ CAT	Sharma and Singh (2010)
Curcumin (200 mg/kg)	Malathion (27 mg/kg)	Mice	4 weeks	↓ LPO activity. ↑ Spermatogenesis. ↑ CAT ↑ Testosterone levels improve maturation abnormalities, intratubular necrosis, and inflammatory infiltrate	Ali and Ibrahim (2018)
Curcumin (200 mg/kg)	Nicotine (0.4 mg/kg)	Mice	28 days	↑ Testosterone levels. ↑ Sperm count. ↑ Sperm motility. ↑ Testis weight	Coşkun et al. (2016)
Curcumin (10, 30, and 60 mg/kg)	Nicotine (0.5 mg/kg)	Mice	28 days	↑ Testosterone. ↑ Sperm count. ↑ Sperm motility. ↑ Testis weight	Jalili et al. (2014)
Curcumin (0, 5, 10, 20, and 40 μM)	Palmitic acid (50–400 µM)	MLTC-1 cells apoptotic model induced by PA	Incubation for 24 h	↑ Cell viability ↓ Caspase three activity ↓ Expression levels of BAX, CHOP, and GRP78. Restored testosterone levels	Chen et al. (2019)
Curcumin (80 mg/kg)	2,3,7,8-tetrachlorodibenzo-*p*-dioxin (TCDD) (50 ng/kg)	Rats	13 weeks	↑ SOD ↑ GSHPx ↓ MDA ↑ Sperm quality	Bulmuş et al. (2013)

**TABLE 3:** The impact of nanocurcumin on reducing chemical-induced reproductive toxicity in males.

Compound/Preparation	Drug and doses	Animal	Duration	Outcome	Reference
Curcumin (100 mg/kg) Nanocurcumin (200 mg/kg)	Aluminium Phosphide (2 mg/kg)	Rats	28 days	↓ MDA ↑ TOS ↑ GSH ↑ TAC ↑ SOD	Ranjbar et al. (2023)
Nanocurcumin	Cadmium (1 mg/kg/body)	Rats	4 weeks	Well-structured seminiferous tubules compared to the degenerated structure in the cadmium-treated group. ↑ PCNA ↓ Caspase 3 ↓ BAX/Bcl-2 ↓ MDA ↓ NO ↑ Sperm Cell Count ↑ Testosterone	Abdel Latif et al. (2020)
Nano-curcumin (nCUR) curcumin (CUR)	Copper sulphate, CuSO_4_ (100 mg/kg)	Rats	7 days	Prevented testicular tissue injury ↑ FSH ↑ LH ↑ Testosterone ↑ StAR ↑ 3β-HSD ↑ CYP17A1 ↑ AR ↓ MDA ↓ NO ↓ NF-κB ↓ iNOS ↓ TNF-α ↓ Bax ↓ Caspase-3 levels ↑ Bcl-2 ↑ Nrf2 ↑ GSH ↑ HO-1 ↑ SOD ↑ CAT	Sarawi et al. (2022)
Curcumin in corn oil (50 mg/kg b.wt) Chitosan-encapsulated curcumin nanoparticles (50 mg/kg b.wt 60 days)	Fenpropathrin (15 mg/kg)	Rats	60 days	↑ Body weight. ↑ Testes weight. ↑ GSI ↑ Sperm count. ↑ Sperm motility. ↑ Serum testosterone. ↑ LH ↑ FSH 17β-estradiol ↑ GSHPx ↑ GSSG/GSH ↑ GSH ↑ StAR, CYP11A1, CYP17A1, CYP19A1, Kiss-1, Kiss-r1 and GLP-1 expression. Normal Histopathological findings	Mohamed et al. (2023)
Curcumin encapsulated in chitosan-protamine nanoparticles (CUR-CPNPs) 250 mg/kg 45 consecutive days	Nicotine (0.6 mg/kg)	Rats	45 days	Improved sperm characteristics. ↓ Oxidative stress. Inhibited inflammation. ↓ Testes and epididymis weight. ↑ Sperm motility. ↑ Sperm viability. ↓ MDA. ↑ GSH ↑ CAT ↑ SOD and degenerative alterations in testicular histopathology	Mongy et al. (2024)

#### 3.1.1 Inorganic compounds

##### 3.1.1.1 Cadmium chloride

Cadmium (Cd), a toxic heavy metal, severely damages the male reproductive system, causing notable testicular injury and disruption of spermatogenesis. Cd-induced oxidative stress, inflammation, blood-testis barrier damage, and sperm concentration/motility/membrane integrity decline (Antar et al., 2023; Bhardwaj et al., 2024). Additionally, Cd has been associated with hormonal dysfunction, such as low testosterone and abnormal gonadotropin secretion, which may contribute to poor fertility in males (Arteaga-Silva et al., 2020; Antar et al., 2023). The fact that cadmium remains in the body for extended periods means it poses a persistent threat to reproductive health. Preventive measures such as regulation and raising awareness are crucial to shield male reproductive functions from cadmium toxicity (Antar et al., 2023).

Exposure to cadmium chloride (CdCl_2_) caused reduced motility, lower sperm count, and decreased serum testosterone levels, along with an increased incidence of sperm deformities in mice. Additionally, exposure to divalent metal chelate (CdCl_2_) led to elevated levels of total superoxide dismutase (T-SOD), glutathione peroxidase (GSH-Px), and glutathione (GSH), as well as increased activity of T-SOD and GSH, while reducing malondialdehyde (MDA) levels. Conversely, curcumin administration improved semen quality, increased testosterone levels, and boosted antioxidant capacity in the mice. Notably, in the Cd-exposed group, curcumin intervention upregulated testicular nuclear factor erythroid 2-related factor 2 (Nrf2) expression and its downstream antioxidant genes (Yang et al., 2019).

Another research also reported that CdCl_2_ caused remarkable oxidative stress and histopathological lesions in the male reproductive system, which may contribute to male infertility. Nevertheless, curcumin counteracted these detrimental effects by reducing oxidative stress and improving histological scores. The results demonstrate that curcumin can potently prevent and ameliorate cadmium-induced reproductive toxicity, which thereby supports its potential as a therapeutic agent in toxicity. In general, the study highlights curcumin’s protective function in the male reproductive system against environmental toxicants (Oguzturk et al., 2012).

Aktas et al. also demonstrated that cadmium exposure resulted in substantial apoptosis and tissue injury in the testes. Interestingly, curcumin treatment significantly inhibited apoptosis and restored the histological integrity of the testis. Furthermore, curcumin administration also enhanced serum testosterone concentration compared with the control group. Results show that curcumin has protective effects against cadmium-induced testicular injury. In general, this study proposes that curcumin could be a therapeutic agent for reducing reproductive toxicity induced by environmental pollutants, such as cadmium (Aktas et al., 2012).

Another study showed that curcumin increased serum antioxidant enzyme levels, which were significantly reduced after cadmium exposure. In this experiment, a controlled experimental design was used with four groups of adult mice: controls, cadmium-treated mice, curcumin-treated mice, and a combination of cadmium and curcumin. Curcumin not only alleviated oxidative stress but also protected the structural integrity of the testicular tissues, as represented by improved histopathology. Moreover, curcumin treatment reduced the levels of inflammatory markers associated with cadmium toxicity. This study elucidates curcumin’s dual role in antioxidant activity and tissue protection, thereby strengthening its potential as a therapeutic against environmental toxins. In general, findings indicate that curcumin plays a comprehensive protective role in male reproductive health compromised by cadmium exposure (Momeni and Eskandari, 2020).

In another study, control and curcumin-treated mice showed normal testicular morphology. Cadmium-treated mice showed severe pathologic changes in the testes, including loss of spermatogenesis and marked germ cell degeneration. Mice pre-treated with curcumin before exposure to cadmium showed only minor damage, with the seminiferous tubules exhibiting normally appearing structures and stages of spermatogenesis. These findings strongly indicate that curcumin significantly reduces cadmium-induced toxicity in the testis (Singh et al., 2012).

##### 3.1.1.2 Copper oxychloride (COC)

The agricultural fungicide copper oxychloride (Cu_2_(OH)_3_Cl or COC) is widely used for its efficacy on several plant diseases. Its toxicity has always been a concern due to its potential to contaminate the environment and affect non-target organisms. It is well established that exposure to COC can induce oxidative stress and have deleterious effects on cellular functions and reproductive tissues in animals. The toxicity of this compound is due to its ROS-generating activity, which compromises cellular integrity and reproductive system function. Experiments have demonstrated that COC can influence seed germination and plant growth, thereby having wider ecological consequences (Paul et al., 2022).

A study has shown that COC exposure results in pronounced testicular injury, manifested in decreased testicular weight, deranged seminiferous tubules, and decreased sperm parameters. In addition, curcumin treatment is highly effective in preventing these toxicities by modulating antioxidant activity and hormonal homeostasis, indicating its potential therapeutic use for reversing copper oxychloride-induced reproductive toxicity (Gad El-Hak and Mobarak, 2020).

##### 3.1.1.3 Lead acetate (Pb(OAc)_2_)

Lead acetate (Pb(OAc)_2_) is a harmful compound known to damage various biological systems, with particular vulnerability observed in the male reproductive system. Studies have shown that in male rats, lead acetate severely impairs sperm quality by reducing motility, viability, and concentration, while increasing the incidence of abnormal sperm. This toxicity is linked to oxidative stress, spermatogenic disorder, hormonal imbalance, decreased testosterone levels, androgen receptor dysregulation, which negatively affect male fertility (Elgawish and Abdelrazek, 2014).

Another study showed that curcumin protects against lead acetate-induced testicular damage in Wistar rats. The current research shows that curcumin is also a natural antioxidant that helps to reduce oxidative stress caused by lead toxicity. It helps maintain testicular structure and function, improving sperm quality and fertility. Curcumin also reduces inflammation, thereby reducing damage to the testicles caused by lead poisoning. In short, the findings indicate that curcumin can protect fertility in areas with heavy metal pollution (Sudjarwo et al., 2017).

##### 3.1.1.4 Lead (Pb), arsenic (As), cadmium (Cd), mercury (Hg), iron (Fe), and copper (Cu)

Another study examined the protective effects of curcumin against the toxic effects of a mixture of heavy metals (lead, arsenic, cadmium, mercury, iron, and copper) and studied their impact for 28 days (Zoheb et al., 2014). Metals exposure was associated with increased oxidative stress markers and a degenerative component of testicular histoarchitecture. This study demonstrated that curcumin treatment mitigates these toxic effects by reducing oxidative stress and preserving testicular morphology. Although certain enzyme activities associated with metal exposure were suppressed, curcumin could counter these impairments. In summary, the findings of this study support the potential of curcumin as a therapeutic option for preventing testicular toxicity caused by heavy metals.

##### 3.1.1.5 Mercuric chloride (HgCl_2_)

Mercuric chloride (HgCl_2_) is a highly toxic environmental contaminant that selectively harms the male reproductive tract. Chronic HgCl_2_ exposure has been reported to produce multiple poisonous effects, such as decreased fertility, decreased levels of testosterone, and impaired sperm function in animal models. Based on the study, exposure to HgCl_2_ reduces sperm count, motility, and morphology, and induces pathological changes in the testes, including degeneration of the seminiferous tubules and Leydig cells (Li et al., 2022).

Another study investigated curcumin’s protective effect against HgCl_2_-induced reproductive toxicity in mice. HgCl_2_ exposure resulted in impaired sexual behavior, reduced fertility, and hormonal disturbances in the offspring. Nevertheless, curcumin administration ameliorated these results, restoring testosterone, progesterone, and other key hormones. Further, curcumin reversed anxiety-like behaviors, indicating its generalized neuroprotective role. The paper describes the therapeutic efficacy of curcumin against environmental toxicants in the context of reproductive health (Mohammad Abu-Taweel, 2020).

##### 3.1.1.6 Nickel-chromium (Ni-Cr) alloys

Nickel-chromium (Ni-Cr) alloys are metallic refractories based on nickel and chromium that are highly resistant to oxidation, exhibit high-temperature stability, and have a wide range of industrial applications, particularly in heating and aerospace. Nevertheless, contact with such alloys, particularly in their elemental form, has raised concerns about their effects on male reproductive function. Studies have shown that chromium, especially in its hexavalent (Cr(VI) form, can affect hormonal homeostasis and spermatogenesis, resulting in sperm quality decline and infertility in men. In addition, nickel exposure has been associated with changes in testosterone and testicular function, and therefore reproductive toxicity. Together, these results highlight the importance of carefully controlling Ni-Cr exposure to avoid potential detrimental effects on male reproductive health (Pereira et al., 2021; Moreira et al., 2023).

Curcumin showed protective effects against nickel and chromium-toxic actions on the rat testicular tissues. These heavy metal exposures inhibited the activity of the major antioxidant enzymes, glutathione peroxidase (GSHPx), superoxide dismutase (SOD), and catalase (CAT), suggesting the presence of oxidative stress. Nevertheless, curcumin treatment almost completely recovered the activities of these enzymes, suggesting its contribution to boosting the antioxidant defense system. Histopathological analysis showed that curcumin prevented Ni-Cr-induced testicular injury, thereby improving tissue structure. Furthermore, curcumin reduces nickel and chromium deposition in testicular tissue, further strengthening its protective effects. In general, the present work highlights curcumin’s potential as a therapeutic agent for metal-induced oxidative injury and its impact on testicular function in the presence of toxicants (El-Amawy et al., 2022).

##### 3.1.1.7 Sodium fluoride (NaF)

It has now been established that fluorides pose a hazard to the male reproductive system. Research indicates that sodium fluoride (NaF) hurts sperm quality, motility, morphology, and capacitation—all of which are critical factors in fertilization (Long et al., 2009; Kim et al., 2015). Furthermore, the effects of fluoride on spermatogenesis and hormonal changes have been documented, including its inhibitory effect on testosterone levels and disruption of growth factor signaling pathways (Kim et al., 2015). Studies involving animal subjects have demonstrated that fertility indicators, such as sperm count and overall mating behavior, are significantly diminished by fluoride administration (El-lethey and Shaheed, 2011). The underlying mechanisms appear to include oxidative stress and alterations in testicular histology, which lead to decreased sperm production and quality (Sánchez-Gutiérrez et al., 2024). In conclusion, the current findings suggest that fluoride represents a substantial threat to male reproductive health, and further research is warranted to comprehend the long-term effects of this chemical on male fertility and potential interventions.

Research findings indicate that curcumin at 100 mg/kg body weight protects against fluoride-induced reproductive damage in mice. Such protection is associated with its antioxidant activity and its ability to inhibit apoptosis, thereby limiting fluoride-related reproductive toxicity in men (Chaithra and Sarjan, 2020).

##### 3.1.1.8 Sodium arsenite (NaAsO_2_)

Sodium arsenite (NaAsO_2_), a toxic inorganic compound, adversely affects the male reproductive system. It decreases sperm concentration, motility, and viability while elevating oxidative stress. These harmful effects are linked to disruptions in testicular function and hormonal imbalances, potentially resulting in reduced male fertility (Souza et al., 2023).

Curcumin supplementation could protect against NaAsO_2_-induced derangement in various sperm quality parameters in adult male mice. Compared with the control group, NaAsO_2_-exposed mice exhibited significantly lower sperm counts, motility, viability, normal morphology, and acrosomal integrity. In addition, the combined treatment of curcumin and NaAsO_2_ was found to substantially reverse the harmful effects on sperm parameters to near normal levels, similar to those in the control group. However, a single application of curcumin alone did not cause a significant difference in these sperm parameters compared with the control and curcumin plus NaAsO_2_ groups. The study concluded that curcumin could mitigate some of the toxic effects of NaAsO_2_ on sperm parameters in adult male mice (Momeni and Eskandari, 2016).

##### 3.1.1.9 Sodium metabisulfite (Na-MBS)

Sodium metabisulfite (Na_2_S_2_O_5_, or NaMBS) is an inorganic compound commonly used for preservation and disinfection, and as an antioxidant in the food and pharmaceutical industries (Ahmadi et al., 2018). Studies show that exposure to NaMBS is capable of having an impact on the male reproductive system. Animal studies have demonstrated that it induces oxidative stress and hormonal changes, which may affect spermatogenesis and fertility (Shekarforoush et al., 2015). These results suggest that greater caution should be exercised when using Na-MBS, particularly in food items consumed by males.

According to Reza Mahmoudi et al., Na-MBS at intermediate (7 mg/kg/day) and high (70 mg/kg/day) doses caused significant histological damage in testicular tissue (Mahmoudi et al., 2017). The researchers reported a 25%–40% reduction in seminiferous tubule volume, epithelial thickness, and tubule length, along with a 15%–20% increase in connective tissue volume. Losses in spermatogonia, spermatids, Sertoli cells, and Leydig cells were recorded at 19%–36% for the intermediate dose and 41%–57% for the high dose (Mahmoudi et al., 2017).

However, on administration of Na-MBS with curcumin at a dose of 100 mg/kg/day, all those morphological changes in the testes as mentioned above were alleviated dramatically in comparison to Na-MBS-treated rats without curcumin treatment. This suggests that curcumin, a polyphenolic compound derived from turmeric, may potentially prevent the adverse effects of Na-MBS on male reproductive health (Lonare et al., 2016; Mahmoudi et al., 2017).

##### 3.1.1.10 Titanium dioxide nanoparticles (nTiO_2_)

Titanium dioxide nanoparticles (nTiO_2_) are nanomaterials with a wide range of industrial applications, including cosmetics, food packaging, and photocatalysis, owing to their photocatalytic activity and chemical inertness. According to the present research state, nTiO_2_ has the potential to cause male reproductive toxicity, promoting oxidative stress, attenuating sperm motility, and changing the hormones, causing fertility reduction (Habas et al., 2021). These toxicities are associated with inflammation and endocrine system impairment; thus, further research on the reproductive toxicity of nTiO_2_ is warranted (Johari and Asghari, 2015; Lange et al., 2020).

Karimi et al. show the protective role of curcumin against testicular toxicity induced by nTiO_2_ in mice (Karimi et al., 2019). nTiO_2_ exposure has been demonstrated to have severely damaging effects on the male reproductive system, such as reduced testicular weight, low testosterone level, impaired sperm quality, and histological abnormalities of the seminiferous tubules. These effects are likely the consequence of nTiO_2_’s uptake by various testicular cells, including spermatids, Sertoli cells, and Leydig cells, thereby inducing oxidative stress and inflammation. Pretreatment with curcumin, noted for its antioxidant and anti-inflammatory nature, effectively mitigates the damaging effects of nTiO_2_ on the testes. Intervention with curcumin before exposure to nTiO_2_ significantly increased testicular mass, morphometric variables, Johnsen score, serum testosterone concentrations, and histological parameters, suggesting curcumin’s protective effects on testicular structure and function.

Curcumin’s protective effects are attributable to its potent antioxidant and anti-inflammatory properties. Curcumin has been found to scavenge free radicals, prevent lipid peroxidation, and modulate antioxidant enzymes, thereby attenuating oxidative stress-mediated damage to testicular cells. In addition, curcumin can mediate the inflammatory cascade by inhibiting NF-κB and reducing the expression of pro-inflammatory cytokines and transcription factors involved in testis toxicity via nTiO_2_.

The findings of this study suggest valuable practical implications for the potential of curcumin as a therapeutic to protect the male reproductive system against nTiO_2_-induced toxicity. However, further research is needed to elucidate the precise mechanisms of its protective action and to confirm its efficacy in human models.

#### 3.1.2 Organic compounds

##### 3.1.2.1 Cypermethrin and deltamethrin

Cypermethrin and deltamethrin are synthetic pyrethroid insecticides used in many countries to control pests. That affects insects' nervous systems, leading to paralysis and death. Both compounds have been incorporated into many insecticide products that target specific pests, such as mosquitoes and other agriculturally important insects, and used for indoor residual spraying and for outdoor vector control of vector-borne diseases, including malaria and dengue fever. Some of these insecticides have been reported to have developed resistance in certain populations/stocks; therefore, further research on their effectiveness and safety in pest management systems is warranted (Sathantriphop et al., 2020).

One study reported that male Wistar rats exposed to cypermethrin and deltamethrin experienced severe reproductive toxicity and oxidative damage. The findings also showed that dietary antioxidants such as curcumin and quercetin effectively mitigated these harmful outcomes. The upregulation of pituitary-gonadal hormones and steroidogenic enzymes, including 3β-hydroxysteroid dehydrogenase (3β-HSD) and 17β-hydroxysteroid dehydrogenase (17β-HSD), was significantly associated with the protective effects of these antioxidants. Using curcumin and quercetin together produced greater protective effects than using them separately. These findings suggest that adding both substances to a regular diet could help reduce reproductive harm associated with exposure to these insecticides (Sharma et al., 2018).

##### 3.1.2.2 Di-*n*-butyl phthalate (DBP)

Di-*n*-butyl phthalate (DBP) is one of the widely used organic plasticizers in the manufacture of flexible plastics, especially polyvinyl chloride (PVC). It is also considered an endocrine-disrupting chemical, and some literature has found its link with negative impact on male fertility, including low testosterone and reduced sperm production. As noted earlier, DBP has been shown to increase oxidative stress and minimize testosterone by inhibiting steroidogenic enzymes, including CYP11A1 and HSD3β2, in human cell lines and animal models. In addition, long-term reproductive effects have also been found to be mediated by DBP; the study establishes that DBP carries the long-term impact of male fertility on exposure. Based on these data, it is also clear that there is a need to regulate the use of, and to look for alternatives to, DBP-containing substances due to health risks to both humans and animals (Källsten et al., 2022a, 2022b).

Curcumin has been found to enhance a protective mechanism involving the specificity protein 1 (SP1)/Peroxiredoxin 6 (PRDX6) pathway, thereby mitigating DBP-induced testicular damage. Research indicates that DBP promotes ferroptosis in testicular tissue by elevating SP1 levels, which, in turn, increase PRDX6, a known negative regulator of ferroptosis. The DBP-induced ferroptotic process in testicular cells can be counteracted through either PRDX6 overexpression or curcumin treatment. In rat studies, concurrent administration of DBP and curcumin reduced DBP-induced testicular ferroptosis. This research underscores the potential of curcumin to reinforce self-protective mechanisms in testicular tissue and mitigate DBP-induced ferroptosis, offering a promising therapeutic strategy to safeguard testicular health against DBP-related harm (Bu et al., 2023).

Another study investigates the protective effects of curcumin and kolaviron against DBP-induced testicular toxicity. DBP exposure caused notable decreases in the testicular weight ratio and further oxidative stress, as evidenced by increased MDA levels. Curcumin and kolaviron treatment significantly increased testicular mass and reduced MDA levels, suggesting reduced oxidative damage. Moreover, the two compounds increased antioxidant enzyme activities (i.e., SOD and CAT), whereas DBP treatment depressed them. The findings suggest that curcumin and kolaviron have the potential to serve as effective therapeutic agents for mitigating reproductive toxicity induced by environmental contaminants. In general, research indicates that natural products may prevent chemical-induced reproductive damage (Farombi et al., 2007).

##### 3.1.2.3 Di(2-ethylhexyl) phthalate (DEHP)

Di(2-ethylhexyl) phthalate (DEHP) is a widely used plasticizer, synthesized from phthalic acid and primarily incorporated into plastic products such as PVC. While it improves the flexibility and durability of plastics, DEHP is also recognized as an endocrine-disrupting chemical that can interfere with hormonal balance in humans and animals.

Evidence shows that DEHP exposure is harmful to the male reproductive system. The evidence from experiments shows that prenatal DEHP exposure is associated with reduced fertility, abnormal sperm quality, and endocrine disruptor effects in male offspring. Exposure to DEHP has been shown to reduce sperm count and motility, induce atrophy in reproductive organs such as the testes, and lower testosterone production (Barakat et al., 2020; Chang et al., 2022). Additionally, these effects can be transgenerational, with their impact extending beyond the directly exposed population to subsequent generations (Radke et al., 2018). The underlying pathophysiology involves oxidative stress and disruption of normal endocrine signaling pathways, both of which ultimately lead to reproductive dysfunction (Radke et al., 2018).

At low concentrations (1–50 μM), curcumin was shown to enhance sperm motility *in vitro* significantly and to reduce DEHP-induced impairment of testicular function *in vivo*. Curcumin treatment reversed several semen quality parameters, including sperm counts and sperm morphology, and restored testicular damage caused by DEHP exposure. This protective activity is attributed to curcumin’s antioxidant properties, which decrease oxidative stress and inflammation induced by DEHP toxicity, thereby contributing to a favorable reproductive outcome in DEHP-exposed males (Głombik et al., 2014).

##### 3.1.2.4 D-galactose

D-galactose is a monosaccharide sugar, also known as “brain sugar,” and is implicated in several critical biological processes, one of which is the synthesis of glycoproteins in nerve tissue. Likewise, high dosages of D-galactose have also been associated with detrimental outcomes on the male reproductive system, particularly on sperm density and mobility in rodents. Additionally, D-galactose may induce oxidative stress and accelerate aging mechanisms, potentially exacerbating reproductive system issues (Parameshwaran et al., 2010).

Yousefi Taba reported that curcumin supplementation has beneficial effects in the galactose-induced aging mouse model (Oguzturk et al., 2012). Administration of curcumin at doses of 25, 50, and 100 mg/kg for 14 days reduced the number of apoptotic cells (TUNEL positive) in the testes compared to the D-galactose group. The epididymal weight index, which was significantly reduced in the D-galactose group, was restored to near-normal levels in the curcumin-treated groups. Histological changes in the testes indicated that D-galactose administration protects the germinal epithelium from reductions, edema, and complete loss of germinal cells in some spermatogenic tubes. On the contrary, curcumin 1 (25 mg/kg) and curcumin 3 (100 mg/ kg) indicated nearly normal morphology of spermatogenic tubules with only minimal shrinkages of germ cells. In comparison, curcumin 2 (50 mg/kg) caused edema and congestion. In addition, 100 mg/kg curcumin supplementation for 2 weeks has a more favorable effect on biochemical parameters and sperm characteristics, and induces less apoptosis in testicular tissue in aged mice.

The outcomes of these studies have established the benefits of curcumin in reducing age-related changes in testicular lesions, apoptosis, and the index of reproductive organ weight. With the dose-graded effects demonstrated, particularly at the 100 mg/kg dose, a dose-response relationship warrants examination. Subsequent analysis can also evaluate curcumin’s protective effects on male reproductive organs and may extend research on age-related male reproductive dysfunction in humans.

##### 3.1.2.5 Hydrogen peroxide (H_2_O_2_)

Hydrogen peroxide (H_2_O_2_) is a ROS that damages the male reproductive system. An increase in H_2_O_2_ concentration is the primary driver of oxidative stress, which, in turn, reduces sperm viability and motility and causes loss of membrane integrity. In particular, higher concentrations of H_2_O_2_ (200 μM) have been reported to minimize sperm penetration through cervical mucus and to inhibit capacitation, a process essential for fertilization. In addition, H_2_O_2_ exposure induces apoptotic signaling responses, resulting in greater reductions in sperm survival and motility. These findings demonstrate the toxic action of oxidative stress towards male fertility (Pujianto et al., 2021; Hussain et al., 2023).

In another investigation, researchers evaluated the effect of dietary curcumin supplementation on H_2_O_2_-induced oxidative damage in the testes of breeder roosters. The study design involved grouping the roosters and assigning each group a specific diet and treatment. Results showed that curcumin supplementation significantly improved sperm quality, enhanced testicular function, protected against H_2_O_2_-induced oxidative damage, and increased antioxidant enzyme activity. Curcumin also reversed adverse effects on gene expression related to spermatogenesis and antioxidant pathways, restored protein expression, and modulated apoptotic pathways. Curcumin dietary supplementation ameliorates oxidative damage and reproductive aging in sows by inducing the Nrf2 signaling pathway and promoting anti-apoptosis (Wu et al., 2023).

##### 3.1.2.6 Imidacloprid (IM)

Imidacloprid (IM) is a neonicotinoid insecticide used in agricultural fields and exhibits neurotoxicity in insects. Findings reveal that IM exposure has adverse effects on male gonadal toxicity and testosterone levels that shrink testes, as well as destroy seminiferous tubules, which may affect fertility due to cell stress and damage in reproductive cells. These findings confirm the need to sharpen the understanding of the effects of IM on male reproductive health in the long run.

According to Milindmitra Lonare et al., curcumin may reduce the toxic effects of IM on the male reproductive system in Wistar rats (Lonare et al., 2016). IM treatment had a profound impact on sperm characteristics, namely, motility, total epididymal sperm count, live sperm count, and sperm abnormality. It was noted that IM exposure causes a decrease in testicular and circulating testosterone levels, together with the 3β-HSD and 17β-HSD activities in the testis. In this case, IM treatment led to increased oxidative stress markers, such as lipid peroxidation (LPO), and decreased antioxidants, including SOD, GSH, glutathione S-transferase (GST), and GSHPx. Histopathological analysis indicated the alterations in the testis and epididymis of IM-treated rats of reproductive toxicity and oxidative stress biomarkers. In this context, curcumin at a dose of 100 mg/kg significantly alleviated the effects of IM on the diagnostic values of reproductive toxicity parameters and oxidative stress biomarkers, and restored altered testicular histopathology. Curcumin showed the possibility to potentially protect against IM-generated male reproductive toxicity through antioxidant, anti-inflammatory, and the ability to regulate overall reproductive system function.

Milindmitra Lonare et al. demonstrated that curcumin could alleviate the toxic effects of IM on the male reproductive system in Wistar rats (Lonare et al., 2016). Sperm motility, total epididymal sperm count, live sperm count, and abnormal sperm were significantly decreased due to IM treatment. Additionally, it was found that IM exposure reduced the testicular and plasma levels of testosterone as well as 3β-HSD and 17β-HSD activities in the testis. In this case, IM treatment resulted in upregulation of oxidative stress markers, such as LPO, and in reduced antioxidant levels, including SOD, GSH, GST, and GSHPx. The histopathological examination revealed changes in the testis and epididymis of IM-treated rats. Curcumin showed protective potential against reproductive toxicity induced by intramuscular injection in men, mainly through its antioxidant, anti-inflammatory, and regulatory effects on the reproductive system. In Wistar rats, co-administration of curcumin (100 mg/kg) with intramuscular injection effectively reduced oxidative stress markers, improved histopathological changes, and restored normal testicular function, thereby reducing adverse effects on reproductive parameters (Lonare et al., 2016).

##### 3.1.2.7 Lindane

Lindane (gamma-hexachlorocyclohexane, γ-HCH), an organochlorine insecticide, has long been widely used in agriculture and forestry and is now prohibited in most countries due to its persistence and toxicity, thereby imposing a significant burden on the environment and human health. It is highly lipophilic, leading to bioaccumulation in living organisms, and is also associated with several health problems, including a potential carcinogenic effect (Sang et al., 1999).

Sharma and Singh investigated the alleviating effect of curcumin against lindane-induced reproductive toxicity. According to this study, curcumin is a highly effective agent for mitigating lindane-induced toxicity to male reproductive function. It emphasizes curcumin as an antioxidant, a pivotal mechanism for counteracting lindane toxicity. Various treatment regimens, including pretreatment and post-treatment with curcumin, showed positive outcomes. Research suggests that curcumin may be a promising therapeutic agent for mitigating the effects of environmental toxicants. These data underscore the potential role of curcumin in restoring reproductive health under chemical exposure. More generally, the present study provides valuable information about approaches for mitigating male reproductive toxicity caused by injurious chemicals (Sharma and Singh, 2010).

##### 3.1.2.8 Malathion

Malathion, an organophosphate insecticide, is used in agriculture to protect crops and control disease vectors that affect humans and animals. It is a cholinesterase inhibitor that interferes with the central nervous systems of insects and other organisms. Honeybees are particularly sensitive to malathion, which is moderately hazardous to mammals and dangerous to aquatic organisms, has low environmental persistence, and poses minimal risk of groundwater contamination (Wilson, 2003).

In another experiment, co-treatment with curcumin and malathion resulted in reduced LPO and enhanced spermatogenesis compared with malathion alone. Moreover, curcumin restored antioxidant enzyme levels, including CAT and SOD, that malathion had suppressed. In addition, malathion has been found to cause a decrease in testosterone levels, but curcumin increased them again. This shows that curcumin ameliorated the pathological changes in the testis, including intratubular necrosis, inflammatory infiltrate, and maturation abnormalities, induced by malathion. These findings indicate that curcumin may be a therapeutic agent against the adverse effects of organophosphate pesticides on the male reproductive system (Ali and Ibrahim, 2018).

##### 3.1.2.9 Palmitic acid

Palmitic acid (PA) is a saturated fatty acid that is extensively found in animal fat as well as vegetable oil. From the existing literature, it was established that high levels of PA are toxic to male fertility through the disruption of the blood-testis barrier and through the induced apoptosis of Leydig cells that determine testosterone synthesis. Particularly, PA exposure has been demonstrated to increase endoplasmic reticulum (ER) stress and subsequent spermatogenic toxicity and reduced testosterone levels that are associated with male infertility (Chen et al., 2019; Mayerhofer et al., 2020; Ge et al., 2022).

Curcumin can prevent PA-induced apoptosis of testicular Leydig cells via ER stress. Chen et al. demonstrate that PA promotes apoptosis via ER stress pathways and that curcumin is a potent inhibitor of these pathways (Lonare et al., 2016). Curcumin not only alleviates ER stress but also inhibits apoptosis, thereby protecting Leydig cell integrity. These findings suggest a potential therapeutic role for curcumin in treating lipid-induced cellular dysfunction. In summary, this study’s findings suggest that curcumin may help reduce PA-induced testicular damage.

##### 3.1.2.10 2,3,7,8-tetrachlorodibenzo-*p*-dioxin

2,3,7,8-tetrachlorodibenzo-*p*-dioxin (TCDD) is a highly toxic compound that belongs to the group of polychlorinated dibenzo-*p*-dioxins. TCDD is often recovered as a by-product of uncontrolled combustion of organic material and is associated with many industrial processes, including herbicide production, similar to Agent Orange. Exposure to TCDD has been linked to reproductive health issues such as poor sperm quality, endocrine disruption, and reduced fertility (Zhang et al., 2021). Specifically, TCDD can disrupt endocrine activities by blocking testosterone synthesis and, in turn, have adverse effects on sex organ development. Repeated exposure may lead to testicular atrophy and reduced sperm motility, many of which may have detrimental effects on male reproductive function and sperm quantity (fertility) (National Toxicology Program, 1982).

The possible protective role of curcumin against somatic effects induced by TCDD in male rats is investigated. Curcumin dosing reduced MDA concentration and, through direct effects on antioxidant enzymes, improved antioxidant status. It also counteracted the inhibition of body weight gain resulting from TCDD treatment. Furthermore, curcumin reversed the TCDD-induced reduction in reproductive performance (sperm density and swim-up velocity). Therefore, curcumin can be attributed to its biomedical applications in the management of TCDD-induced toxicity (National Toxicology Program, 1982).

#### 3.1.3 Nanoformulations

##### 3.1.3.1 Aluminum phosphide (AlP)

Aluminum phosphide (AlP or PH3) is a highly toxic inorganic compound, primarily used as a fumigant and pesticide in agriculture. AlP releases phosphine upon contact with moisture, a poisonous gas. Its mechanism includes cellular hypoxia secondary to mitochondrial dysfunction, resulting in severe cardiovascular consequences, including hypotension and arrhythmias. Signs of poisoning may include nausea, vomiting, and respiratory inability, as well as pulmonary edema and shock. Due to its toxicity and acute effects, AlP poses a significant health threat, particularly in agricultural areas where it is widely used (Gurjar et al., 2011).

Research in rats exposed to AlP toxicity showed that co-treatment with curcumin and nanocurcumin significantly reduced oxidative damage in the testes, increased antioxidant levels, and improved sperm quality compared to the control group. These results suggest that curcumin, especially in its nanoform, may act as a potent protective agent against oxidative stress-related impairments in sperm parameters and overall testicular function (Ranjbar et al., 2023).

##### 3.1.3.2 Cadmium chloride (CdCl_2_)

Abdel Latif et al. demonstrated that nanocurcumin ameliorates testicular toxicity induced by CdCl_2_ in male rats (Abdel Latif et al., 2020). The findings include enhanced histological features of the seminiferous tubules, increased PCNA staining, reduced levels of caspase 3 and the BCL-2-associated X protein (BAX)/B cell lymphoma-2 (Bcl-2) ratio, elevated levels of MDA and NO, and increased sperm concentration and testicular testosterone levels. Therefore, the results of this study suggest that nanocurcumin may have therapeutic potential against the toxic effects of environmental pollutants, such as cadmium, on testicular tissue. By improving cellular structural organization and function, reducing oxidative damage, and enhancing reproductive variables, nanocurcumin demonstrates its potential to promote reproductive health. However, further studies are needed to explore its mechanisms of action and its applicability to human health, particularly for individuals exposed to heavy metals.

##### 3.1.3.3 Copper sulfate (CuSO_4_)

Copper sulfate (CuSO_4_) is an essential component in various biological processes; however, excessive doses can be toxic to male reproductive organs. Research findings suggest that CuSO_4_ has a detrimental effect on spermatogenesis, primarily through oxidative stress and autophagy, resulting in hormonal imbalances and reduced sperm quality. MDA levels increase in response to CuSO_4_ exposure, thereby enhancing oxidative stress and affecting testicular function and sperm quality (Roychoudhury). In addition, CuSO_4_ has been shown to reduce testosterone, luteinizing hormone (LH), and follicle-stimulating hormone (FSH) levels in affected individuals, thereby adversely affecting reproductive health (Sarawi et al., 2022; Li and Wang, 2023). Hormonal morphological changes are observed at the histological level, characterized by alterations in testicular size and weight, as well as increased apoptosis and caspase-3 levels (Liu et al., 2016; AL-Musawi et al., 2022). Moreover, CuSO_4_ activates autophagy through the AMP-activated protein kinase(AMPK)-mammalian Target of Rapamycin (mTOR) signaling that can either prevent or aggravate testicular injury depending on the circumstances (Guo et al., 2022). Although CuSO_4_ is present in minute quantities, its effects on the male reproductive system demonstrate how copper can cause reproductive diseases when its levels are poorly regulated in the environment and in food products.

A study strongly supports the deleterious effect of CuSO_4_ on testicular tissue, pituitary-gonadal axis, and steroid hormone synthesis, while also highlighting the protective benefits of curcumin (CUR) and nanocurcumin (nCUR). Both CUR and nCUR prevented copper-associated testicular damage, reduced oxidative stress, inflammation, and apoptosis, and increased cell proliferation, circulating sex hormones, and steroid production. In mice, these protective results were associated with activation of the Nrf2/HO-1 signaling pathway and improved antioxidant defense. nCUR showed greater efficacy than curcumin, likely due to its improved physicochemical properties. Overall, nCUR shows strong potential as a defense against CuSO_4_-induced reproductive toxicity, although further studies are needed to elucidate its mechanisms of action (Sarawi et al., 2022).

##### 3.1.3.4 Fenpropathrin (FNP)

Fenpropathrin (FNP) is a manufactured pyrethroid insecticide widely used in agriculture to manage mites on fruit and vegetable crops. FNP kills insects by blocking sodium channels in their nerves, leading to paralysis and eventual death. Recent research has highlighted the potential harm to the heart. Fenpropathrin was found to increase heart rate and alter blood circulation in zebrafish. Additionally, FNP has been reported to cause neurological damage in mammals, giving rise to its use having questionable safety for human health and the environment. The acceptable daily intake (ADI) level was set at 0–0.03 mg/kg body weight by the regulatory reviews. This is to ensure that agricultural practices are well managed to minimize the health risks associated with their application (Xiong et al., 2016; Saputra et al., 2023).

A recent investigation evaluated the adverse impacts of FNP on the male rat reproductive system. It contrasted the effects of curcumin and curcumin-encapsulated chitosan nanoparticles (CS.CUR.NPs) on these toxic effects. Exposure to FNP induced oxidative stress, sperm abnormalities, decreased hormone levels, and altered gene expression associated with spermatogenesis and steroidogenesis. Administration of CUR and CS.CUR.NPs demonstrated remedial effects on semen quality, hormone levels, antioxidant capacity, and gene expression. CS.CUR.NPs exhibited properties superior to those of CUR. The findings of this research indicate that encapsulating curcumin in chitosan nanoparticles enhances its efficacy in mitigating the toxic effects of FNP (Mohamed et al., 2023).

##### 3.1.3.5 Nicotine

Nicotine is an alkaloid that is found in tobacco and mainly causes addiction in people. This is especially true in the male reproductive system, where it negatively impacts sperm production and mobility and increases DNA fragmentation in sperm cells. Nicotine exposure impairs hormonal signaling, reduces testosterone levels, and increases oxidative stress, all of which negatively affect male fertility and reproductive health (Oyeyipo et al., 2011; Firouzabadi et al., 2025).

Nicotine-induced reproductive disorders in male rats can be ameliorated using curcumin-loaded chitosan-protamine nanoparticles. One study demonstrated that curcumin encapsulated in chitosan-protamine nanoparticles has a potent antitumor effect in breast cancer cells, particularly MCF-7 cells. The synthesized nanoparticles had a mean diameter of 200 nm, a zeta potential of +26.66 mV, and high EE and LC. Molecular analysis of cell-based assays revealed decreased cell viability, reduced levels of NF-κB, TNF-α, and IL-6, and downregulation of the anti-apoptotic gene Bcl-2. In conclusion, curcumin-loaded nanocarriers exhibit potent antitumor activity by downregulating pro-inflammatory cytokines and upregulating apoptotic gene expression, which may be used to ameliorate nicotine-induced reproductive dysfunction in male rats (Mongy et al., 2024).

In another study, various doses of curcumin (10, 30, and 60 mg/kg) were given together with nicotine (0.5 mg/kg) for 28 days. The findings showed that nicotine hurts testicular function by lowering testosterone levels, sperm count, motility, and testicular mass compared with the control group. Nevertheless, higher doses of curcumin significantly improved these reproductive indices. This implies that curcumin can be used to reverse the impacts of nicotine on male fertility (Jalili et al., 2014).

Another study demonstrated that curcumin-treated mice had less damage to spermatogenic, Sertoli, and Leydig cells than mice exposed only to nicotine. The administration of curcumin also resulted in a minor change in testicular structure after 28 days of treatment. Furthermore, curcumin treatment also prevented the reduction of testosterone levels due to nicotine exposure. These results indicate that curcumin could be used as a therapeutic agent for testicular injury associated with nicotine dependency (Coşkun et al., 2016).

## 4 Conclusion

**FIGURE 3:**
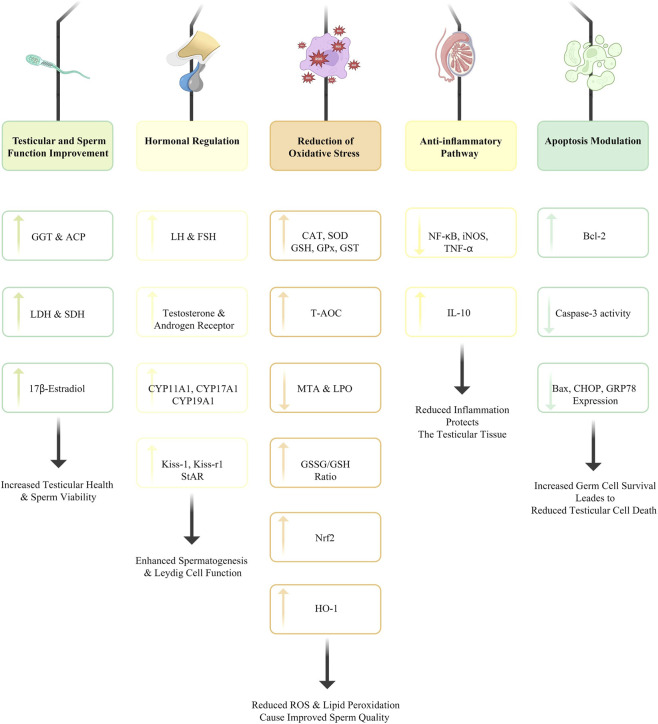
Exploring the protective mechanisms of curcumin in preserving male reproductive health against chemical-induced toxicity.

Curcumin offers a range of protective benefits against drug-induced damage in the male reproductive system by acting as an antioxidant, regulating hormone levels, inhibiting apoptosis, and modulating inflammation. Further research is warranted to clarify the molecular mechanisms behind these effects and to explore curcumin’s potential therapeutic use in reproductive toxicology (Figure 3).

Although most of the included studies demonstrated protective effects of curcumin at doses ranging from 50 to 200 mg/kg in animal models, extrapolating these findings to humans requires careful consideration. Differences in metabolism, bioavailability, and dosing strategies between animals and humans underscore the need for well-designed clinical studies to determine safe and effective human-equivalent doses. Nevertheless, existing evidence suggests that curcumin is generally well tolerated, supporting its potential as a safe adjunctive agent for reducing chemically induced toxicity in the male reproductive system.

Curcumin exhibits potent antioxidant properties, which are crucial mechanisms for attenuating oxidative stress induced by toxicants. It has been shown that curcumin increases the activity of multiple antioxidant enzymes, enhances the concentrations of SOD, CAT, and GSHPx, and increases the levels of the antioxidant GSH. The antioxidant protective effects stabilize LPO and MDA levels, both of which are markers of oxidative stress in testicular tissues.

Curcumin has previously been shown to modulate hormonal profiles by increasing serum concentrations of FSH, LH, and testosterone in male subjects exposed to toxicants such as bisphenol A (BPA). These hormones are also essential for normal spermatogenesis and overall reproductive function. Furthermore, curcumin modulates the steroidogenic acute regulatory protein (StAR) and cytochrome P450 enzymes, such as CYP11A1 and CYP17A1, thereby enhancing steroidogenesis (Riahi et al., 2021; Rasool et al., 2024).

The protective effects of curcumin are also demonstrated for apoptosis regulation of testicular cellular aspects. It has been reported to reduce caspase-3 activity, an effective marker of apoptosis, and to regulate the expression of pro-apoptotic proteins, such as BAX, and anti-apoptotic proteins, such as Bcl-2 (Riahi et al., 2021). Downstream inhibition of the apoptotic signaling pathway contributes to the survival of spermatogenic cell pools under stress from toxic exposures.

Curcumin has additional anti-inflammatory properties by inhibiting the production of pro-inflammatory cytokines, such as TNF-α and iNOS, and by activating NF-κB (Sengul et al., 2023; Walker, 2024). This modulation can be applied to attenuate drug-mediated inflammation damage in the testis by using other pharmacological agents.

In addition, curcumin’s protective properties have been associated with its upregulation of key enzymes involved in detoxification and antioxidant defense. For example, research has shown that the activities of GGT and LDH are upregulated after exposure to curcumin in models of drug-induced toxicity (Al-Bayati and Al-Zubaidi, 2024; Rasool et al., 2024). In addition, curcumin regulates the expression of stress response proteins (e.g., HO-1 and Nrf2) and enhances cellular resistance to oxidative stress (Walker, 2024).

TAC and TOS are considered valid biochemical indicators for evaluating oxidative homeostasis in the reproductive system. Evidence indicates that curcumin supplementation increases TAC and reduces TOS, thereby creating an environment conducive to optimal spermatogenesis (Rasool et al., 2024).

Curcumin and nanocurcumin have been investigated in pharmacogenomics, specifically examining how genetic polymorphisms relate to individual patient responses to the drugs. Curcumin has been shown to bind to a range of molecular targets involved in drug metabolism, such as cytochrome P450 enzymes and transport proteins, and to modulate pharmacokinetics and pharmacodynamics. The bioavailability of curcumin is relatively low owing to poor absorption, rapid metabolism, and rapid elimination. Nonetheless, nanocurcumin selectively improves the solubility and stability of curcumin, thereby enhancing both drug solubility and drug stability, and ultimately improving bioavailability and therapeutic effect. Evidence of the genetic modulation allowing nanocurcumin to impact drug metabolism and transport genes has been reported in the literature, opening the possibility of personalized therapeutic strategies based on curcumin’s protective effects for a series of diseases affecting the male reproductive system (Pal et al., 2015; Maiti et al., 2016; Subramani et al., 2020; Bhardwaj et al., 2024).

Curcumin and nanocurcumin exhibit unequal protective effects against chemical toxicity in the male reproductive system due to differences in their physicochemical properties. Curcumin, as a substrate, exhibits antioxidant and anti-inflammatory properties; however, its poor bioavailability limits its protective efficacy. In comparison, nanocurcumin exhibits increased intracellular uptake and a gradual release in the biological system, demonstrating significantly greater protective activity against oxidative stress and cytotoxic agent-induced cellular damage. According to the research, nanocurcumin not only exhibits greater efficacy than curcumin in reducing oxidative stress markers but also enhances sperm quality and hormone levels in animal models of toxicity. This increased efficacy is attributed to enhanced cellular uptake and improved nanocurcumin tissue retention, resulting in a stronger response to chemical-induced reproductive toxicity (Pal et al., 2015; Maiti et al., 2016; Subramani et al., 2020).

In summary, while there is growing evidence supporting curcumin’s protective role against chemical toxicity in animal models, comprehensive human studies are still needed to validate these effects. The existing animal research provides a foundation for future clinical trials exploring curcumin’s potential as a therapeutic agent to protect male reproductive health from chemical-induced damage.

## Glossary

3β-HSD: 3β-hydroxysteroid dehydrogenase
17β-HSD: 17β-Hydroxysteroid dehydrogenase
ACP: Acid phosphatase
ADI: Acceptable daily intake
AlP: Aluminum phosphide
AMPK: AMP-activated protein kinase
AR: Androgen receptor
BAX: BCL-2-associated X protein
Bcl-2: B cell lymphoma-2
BPA: Bisphenol A
CAT: Catalase
CHOP: CCAAT/enhancer binding protein homologous protein
ER: Endoplasmic reticulum
FNP: Fenpropathrin
FSH: Follicle-stimulating hormone
G6PD: Glucose-6-phosphate dehydrogenase
GGT: Gamma-glutamyl transferase
GRP78: Glucose-regulated protein 78
GSHPx: Glutathione peroxidase
GSH: Glutathione
GST: Glutathione S-transferase
HO-1: Heme oxygenase-1
iNOS: Inducible nitric oxide synthase
LDH: Lactate dehydrogenase
LH: Luteinizing hormone
LPO: Lipid peroxidation
MDA: Malondialdehyde
mTOR: mammalian Target of Rapamycin
NF-κB: nuclear factor-kappa B
NO: Nitric oxide
Nrf2: Nuclear factor erythroid 2-related factor 2
Prdx6: Peroxiredoxin 6
PRISMA: Reporting Items for Systematic Reviews and Meta-Analyses
PVC: Polyvinyl chloride
ROS: Reactive oxygen species
SDH: Sorbitol dehydrogenase
SOD: Superoxide dismutase
SP1: specificity protein1
StAR: Steroidogenic acute regulatory protein
TAC: Total antioxidant capacity
TBARS: Thiobarbituric acid-reactive substances
TNF-α: Tumor necrosis factor-alpha
TOS: Total oxidant status
T-SOD: Total superoxide dismutase.
